# An Accurate Prostate Cancer Prognosticator Using a Seven-Gene Signature Plus Gleason Score and Taking Cell Type Heterogeneity into Account

**DOI:** 10.1371/journal.pone.0045178

**Published:** 2012-09-28

**Authors:** Xin Chen, Shizhong Xu, Michael McClelland, Farah Rahmatpanah, Anne Sawyers, Zhenyu Jia, Dan Mercola

**Affiliations:** 1 Department of Pathology and Laboratory Medicine, University of California Irvine, Irvine, California, United States of America; 2 Department of Genetics and Geneticist Botany and Plant Sciences, University of California Riverside, Riverside, California, United States of America; 3 Vaccine Research Institute of San Diego, San Diego, California, United States of America; Van Andel Institute, United States of America

## Abstract

One of the major challenges in the development of prostate cancer prognostic biomarkers is the cellular heterogeneity in tissue samples. We developed an objective Cluster-Correlation (CC) analysis to identify gene expression changes in various cell types that are associated with progression. In the Cluster step, samples were clustered (unsupervised) based on the expression values of each gene through a mixture model combined with a multiple linear regression model in which cell-type percent data were used for decomposition. In the Correlation step, a Chi-square test was used to select potential prognostic genes. With CC analysis, we identified 324 significantly expressed genes (68 tumor and 256 stroma cell expressed genes) which were strongly associated with the observed biochemical relapse status. Significance Analysis of Microarray (SAM) was then utilized to develop a seven-gene classifier. The Classifier has been validated using two independent Data Sets. The overall prediction accuracy and sensitivity is 71% and 76%, respectively. The inclusion of the Gleason sum to the seven-gene classifier raised the prediction accuracy and sensitivity to 83% and 76% respectively based on independent testing. These results indicated that our prognostic model that includes cell type adjustments and using Gleason score and the seven-gene signature has some utility for predicting outcomes for prostate cancer for individual patients at the time of prognosis. The strategy could have applications for improving marker performance in other cancers and other diseases.

## Introduction

Prostate cancer is the most frequently diagnosed male cancer and the second leading cause of cancer death in men in the United States [1]. Radical prostatectomy is an effective option when the cancer is localized to the prostate gland [2], [3]. However, at the time of diagnosis it is difficult to determine which patients harbor aggressive disease that will recur after treatments designed to cure and which are indolent and suitable for prophylaxis and other strategies. Recurring disease commonly leads to metastasis, the major cause of prostate cancer death [4], [5]. Therefore, a major current issue in clinical management is determining reliable prognostic indicators that distinguish indolent cancer from those that will recur. Classification systems such as the Kattan nomograms [6], D’Amico classification [7], and CAPRA (Cancer of the Prostate Risk Assessment) score [8] that incorporate the measurement of several preoperative and postoperative clinical markers can be used to predict the probability of recurrence after radical prostatectomy. However, prostate cancer patients with similar clinical and pathological features cannot be differentiated by these classification systems as individual risk is not accurately taken into account. Extensive previous efforts have attempted to identify gene expression changes between aggressive cases and indolent cases [9]–[11]. Standard analytical approaches, such as t-test, significance analysis of microarray (SAM) [12] and linear models for microarray data (LIMMA) [13], have been applied to these studies. Few reproducible and clinically useful prognostic biomarkers have emerged. One reason accounting for such inconsistency across studies might be the heterogeneity in terms of cell composition, *i.e.*, the tissue samples used for assays were usually mixture of various cell types with varying percentages [14]–[16] as well as genetic heterogeneity of the polyclonal and multifocal nature of prostate cancer. Therefore, the observed gene expression changes among samples may be due in part to the difference in cell composition of these samples [16]. Nevertheless, such composition heterogeneity is rarely taken into account in biomarker studies because there has been no straightforward way to deal with such variation through regular gene expression analyses.

Here we investigate whether varying cell type composition plays an important role in the identification of differentially expressed genes. We developed a Cluster-Correlation Analysis model [17] that incorporates a multiple linear regression model to consider cell type composition for samples with known composition. We show that this method may be used to identify differentially expressed genes between biochemical relapse and non-relapse patient samples after prostatectomy. Applying this approach we observed more than three hundred gene expression changes and categorized these into predominantly tumor cell expressed genes or stroma cell expressed genes. We identified a subset of seven tumor cell expressed genes that exhibited the most significant changes and used these to derive a classifier. The classifier was then tested on two independent Data Sets with high accuracy and sensitivity. A classification model combing this seven-gene signature with Gleason sum had even better prediction performance. Our results provide novel insights into the development of prostate cancer prognosis.

## Materials and Methods

### Prostate Cancer Patient Samples and Microarray Analysis

Data Set 1 was used for training. It contains 136 post prostatectomy frozen tissue samples obtained from 82 subjects by written informed consent as approved by the UCI Office Research Administration Institutional Review Board (IRB). The IRB specifically approved this study annually (HS#2005-4806). All tissues were collected at surgery and escorted to pathology for expedited review, dissection and snap freezing in liquid nitrogen. The “top” and “bottom” sections of manually microdissected (see Manual Microdissection) frozen tissues were used for tissue composition determination. The rest sections of manual microdissected frozen tissues were used for RNA preparation and microarray hybridization. The tissue composition (tumor epithelial cells, stroma cells, epithelial cells of BPH and dilated cystic glands) was determined by members of a team of four pathologists three of which are Board Certified while the forth is equivalently certified (UK, FRCP) using methods described previously [15]. The boxplot of tissue percentage data was provided is shown in Figure S3. The resulting Microarray data have been deposited in the Gene Expression Omnibus (GEO) database with accession number GSE8218 [16]. Out of the 136 samples, 80 samples were from biochemical relapsed patients, 50 samples from biochemical non-relapsed patients with follow-up from 3 to 80 months, and 6 samples from normal subjects. Conventional clinical markers such as Prostate Specific Antigen (PSA), post-prostatectomy Gleason sum, age, pathologic stage, were also collected and presented in Table S1 and S2.

Data Sets 2 and 3 are independent test sets. Data Set 2 [GSE25136 [18]] contained 79 samples consisting of 42 biochemical non-relapsed and 37 biochemical relapsed samples. Data Set 3 [GSE3325 [19]] consists of 13 samples classified as 4 benign, 5 primary, and 4 metastatic prostate cancer samples. In our study, we treated the 4 benign and the 5 primary prostate cancer samples as biochemical non-relapse samples and 4 metastatic prostate cancer samples as relapse samples. The microarray platforms for Data Set 2 and 3 are Affymetrix U133A and U133 plus 2.0, respectively. The tissue components information was estimated through CellPred software [16] due to lack of cell type percentage information for the two independent Data Sets. Post prostatectomy Gleason sums, Disease Free Survival Times, age, pathologic stage were collected and presented in Table S1 and S2.

### Statistical Analysis

#### Cluster-Correlation analysis model

We developed a novel Cluster-Correlation (CC) analysis procedure [17] for the determination of differential gene expression in various cell types. The CC analysis is implemented in 2 steps, i.e., an unsupervised cluster step and a correlation step (Figure S1).

The unsupervised cluster step is based on two principal assumptions. Assumption 1, the observed gene expression values such as by an expression array is the sum of the contributions from different types of cells that made up the sample (Eqn. 1).
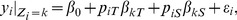
(1)Where *Z_i_* is the cluster indicator for the *i*th sample, *p_iT_* and *p_iS_* are known tumor and stroma percentages [16] for the *i*th sample, *β_kT_* and *β_kS_* are tumor and stroma cell-type coefficients as determined by the multiple linear regression result for the *k*th cluster, and *ε_i_* is the residual error. Each cell-type contribution is in turn due to the product of the percentage of the cell type present and the individual cell type expression coefficient for a given gene. Assumption 2, the individual cell type expression coefficients *β_T_* and *β_S_* for a given gene may vary by the biochemical outcomes of the sample, *e.g.,* biochemical recurrence status. Based on these assumptions, the patient samples form a mixture distribution which can be analyzed with the EM algorithm (Expectation-Maximization) [20]. The EM algorithm finds the optimal solutions through an iterative computation. The results of the EM algorithm are two folds. First, samples were assigned to several clusters (unsupervised) based on the expression values of each gene. Second, we are able to determine the extent of expression of a gene by tumor cells and by stroma cells.

In the correlation step, we selected genes for which relapse and non-relapse cases were well distinguished by the unsupervised clustering procedure. For each gene, we formed a 2×2 contingency table with one dimension as the observed relapse status and the other dimension as the unsupervised clustering result (cluster identity). A Chi-square test was used to calculate p value for each gene (each contingency table). The genes with p-values <0.005 were selected as highly correlated between unsupervised and observed cluster membership.

For the significant genes identified in the correlation step, we determined whether their expression is predominantly expressed in tumor cells and stroma cells. Two restricted models with respect to tumor cells and stroma cells were defined. In the tumor restricted model, we assume only *β_T_* varies with cluster membership. In the stroma restricted model, we assume only *β_S_* varies with cluster membership. The two restricted models were then compared using Bayesian information criterion (BIC) [21]. The model with the smaller BIC score is selected. Differences of 2 or more between two BIC scores is considered as a strong indication favoring one model over another [22].

The CC analysis algorithm and test data set are available on http://www.pathology.uci. edu/faculty/mercola/UCISpecsHome.html and may be applied to expression Data Sets given the knowledge of the cell type distribution.

#### Statistical tools in R

A modified quantile normalization function “REFnormalizeQuantiles” [14] was used to perform normalization for Data Sets 2 and 3 by referencing Data Set 1. Because the probe sets for the U133A platform is the subset of those from the U133 plus 2.0 platform, we carried out the normalization for the common probe sets of the two platforms.

Significant Analysis of Microarray (SAM) [12] of the “siggenes” package, implemented in R, was used to select the most significant genes obtained from the two-step cluster analysis.

Prediction Analysis of Microarray (PAM) [23] of the “pamr” package, implemented in R, was used to develop a prognostic classifier using a training set and the performance of the classifier was tested using independent sets. Data Set 1 was treated as a training set, and Data Sets 2 and 3 were treated as test sets.

An R-based web service, CellPred [16] available at http://www.webarray.org was used to predict the cell composition percentage of Data Sets 2 and 3 in order to identify tumor cell enriched samples for testing of the classifier. Samples for testing were chosen from Data Sets 2 and 3 using the criterion of >50% tumor epithelial cell composition according to CellPred.

#### Immunohistochemistry data analysis

In order to validate the cell type specificity of RNA expression predicted here, we compared the cell type expression intensity, *β_T_*, with the corresponding protein expression in tumor and stroma cells as observed in the Human Protein Atlas (HPA; www.humanprotein.atlas.org). Each HPA antibody was applied to single histology sections from each of three normal subjects and two histology sections from each of 12 prostate cancer patients thus generating three high-resolution images for the normal cases and 24 high-resolution images from the 12 cancer patients. All images were downloaded thereby providing all pixel values of three color channels. The level of protein expression is summarized using the scale: red, strong; orange, moderate; yellow, weak; and white, negative as provided by HPA. Two observers, a board certified pathologist (DAM) and a second observer (XC) further categorized the level of protein expression by adding moderate to strong, weak to moderate, and very weak according to the IHC color intensity and summarized the seven levels using an numeric code: 5, strong; 4, moderate to strong; 3, moderate; 2, weak to moderate; 1, weak; 0.5, very weak; and 0, negative. The protein expression levels in tumor and stroma cells can be estimated based on the numeric code for each image. We collected data for 71 antibodies related to 49 tumor cell expressed genes (no HPA antibodies were available for the remaining 19 genes). We then selected 28 differentially expressed antibodies between normal subjects and prostate cancer patients for the correlation study (antibodies with no protein expression change between normal subjects and prostate cancer patients are considered as non-differentially expressed antibodies). The 28 selected antibodies are related to 23 tumor cell expressed genes. For each antibody, the protein expression level in tumor and stroma is averaged across the 12 patient samples. All 672 IHC observations were used.

## Results

### Development of a Prognostic Classifier

For the Cluster Correlation analysis, we selected 130 arrays of prostate cancer samples obtained from Data Set 1, *i.e*. omitting the remaining six normal samples. We assumed that the EM algorithm of the CC analysis model would categorize the 130 samples into two expression clusters and treated the two expression clusters as putative low risk and high risk groups (*cf.* Figure S1). Then the Chi-square test was performed to measure the association between the putative risk groups and the observed biochemical relapse and non-relapse groups. 324 genes were identified with p-values less than 0.005. The 324 genes were further categorized into 68 predominantly tumor cell expressed genes and 256 predominately stroma cell expressed according to the BIC scores of tumor and stroma restricted models.

In our current study, we focus on investigating the tumor cell expressed genes because the majority of the samples available for independent testing considered below are tumor-enriched samples. The 68 tumor cell expressed genes were considered as candidate genes to develop a prognostic classifier based on their differential gene expression between the observed relapse and nonrelapse groups and the application of SAM. However, it would not be appropriate to perform differential expression analysis of the tumor component directly with all the 130 samples of Data Set 1 because the estimated tissue components showed a large variation of the cell type composition percentage among these samples, including samples with almost exclusively stroma. So we first selected 23 samples with tumor cell percentage greater than 50%. Among 23 selected tumor cell enriched samples, 11 samples are non-relapse samples and 12 samples are relapse samples. Using the 68 genes as input to SAM, we identified the 7 most significant genes between relapse and non-relapse groups where each p value was <0.002 (Table 1). The overall procedure of developing the prognostic classifier is presented as a flow chart in Figure S1.

**Table 1:** Seven-gene signature for prostate cancer prognosis.

Transcript name	Gene	Gene product name	FC
221523_s_at	RRAGD	Ras-related GTP binding D	0.45
214527_s_at	PQBP1	polyglutamine binding protein 1	2.08
208490_x_at	HIST1H2BC///HIST1H2BE///HIST1H2BF///HIST1H2BG///HIST1H2BI	histone cluster 1, H2bg///histone cluster 1, H2bf///histone cluster 1,H2be///histone cluster 1, H2bi///histone cluster 1, H2bc	1.88
207016_s_at	ALDH1A2	aldehyde dehydrogenase 1 family, member A2	0.49
213293_s_at	TRIM22	tripartite motif-containing 22	0.54
209487_at	RBPMS	RNA binding protein with multiple splicing	0.49
221667_s_at	HSPB8	heat shock 22 kDa protein 8	0.44

To validate the prediction accuracy, a PAM-based Seven-gene Prognostic Classifier was generated in order to perform a cross-validation test using the tumor-enriched samples in Data Set 1. For the cross validation, we randomly selected 9 relapse and 8 non-relapse tumor cell enriched samples as a training set leaving the remaining 3 relapse and 3 non-relapse samples as a test set. The PAM-based classifier was then tested on all possible rounds (36300 rounds) of the cross-validation with an average accuracy of 74%, specificity of 72%, and sensitivity of 77%. These results indicate that the Seven-gene Prognostic Classifier has high prediction accuracy, specificity, and sensitivity following the cross validation test and might be efficient for predicting outcomes of prostate cancer patients from independent Data Sets.

### Independent Testing of the Seven-gene Prognostic Classifier

**Figure 1:**
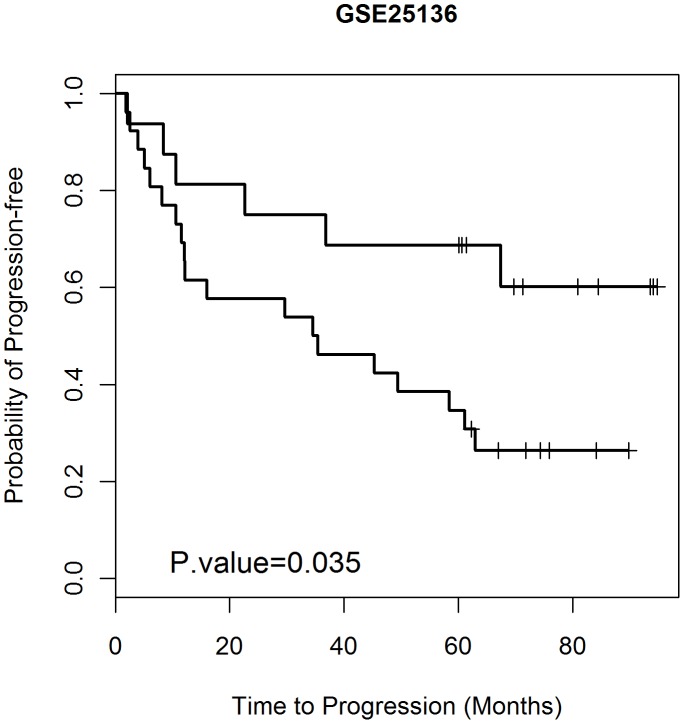
Survival analysis for the seven-gene Classifier. Kaplan-Meier estimates of survival time of 42 independent patients in Data Set 2 (GSE25136) according to the seven-gene Classifier.

A major obstacle in developing clinically useful prognostic profiles for prostate cancer has been a lack of generality across data sets. We therefore tested the Seven-gene Prognostic Classifier on samples drawn from two independent Data Sets (Materials and Methods). However we previously observed that several of the major available expression analysis data sets are very heterogeneous with respect to cell-type composition [16]. Test samples were selected on the basis that they were composed of at least 50% tumor cell content as judged by application of CellPred [16]. Forty two and seven tumor cell enriched samples in Data Sets 2 and 3 respectively met the criterion. Each case was then categorized by PAM using the 7-gene Prognostic Classifier. Table 2 shows the results of the classification. The overall accuracy, specificity, and sensitivity of the two test Data Sets were 71%, 65%, and 76%. To further evaluate the power of the prognostic classifier, we performed Kaplan-Meier survival analysis (Figure 1) (the Kaplan-Meier survival analysis was applied to Data Set 2 only because Disease Free Survival Times is not available for Data Set 3. The comparison shows that the median relapse-free survival of the patients in low risk group defined by the seven-gene prognostic classifier was 35 months. 73% of patients in the high risk group had disease recurrence within 5 years, whereas 63% of patients in the low risk group remained relapse-free for at least 5 years. The estimated hazard ratio for the low risk and high risk group was 2.6 with significant p value of 0.035 (logrank test).

**Table 2:** Comparison of PAM-based gene classifier in two independent tests.

Date Set	Gene classifier	Sensitivity	Specificity	Accuracy
GSE25136	Seven-gene signature	76% (19 of 25)	59% (10 of 17)	69% (29 of 42)
	Bismar gene signature	96% (24 of 25)	0% (0 of 17)	57% (24 of 42)
	Glinsky gene signature 1	56% (14 of 25)	59% (10 of 17)	57% (24 of 42)
	Glinsky gene signature 2	100% (25 of 25)	0% (0 of 17)	60% (25 of 42)
	Glinsky gene signature 3	100% (25 of 25)	0% (0 of 17)	60% (25 of 42)
GSE3325	Seven-gene signature	75% (3 of 4)	100% (3 of 3)	86% (6 of 7)
	Bismar gene signature	50% (2 of 4)	0% (0 of 3)	29% (2 of 7)
	Glinsky gene signature 1	100% (4 of 4)	100% (3 of 3)	100% (7 of 7)
	Glinsky gene signature 2	100% (4 of 4)	0% (0 of 3)	57% (4 of 7)
	Glinsky gene signature 3	100% (4 of 4)	0% (0 of 3)	57% (4 of 7)
GSE25316	Seven-gene signature	76% (22 of 29)	65% (13 of 20)	71% (35 of 49)
+ GSE3325	Bismar gene signature	90% (26 of 29)	0% (0 of 20)	53% (26 of 49)
	Glinsky gene signature 1	62% (18 of 29)	65% (13 of 20)	63% (31 of 49)
	Glinsky gene signature 2	100% (29 of 29)	0% (0 of 20)	59% (29 of 49)
	Glinsky gene signature 3	100% (29 of 29)	0% (0 of 20)	59% (29 of 49)

**Figure 2:**
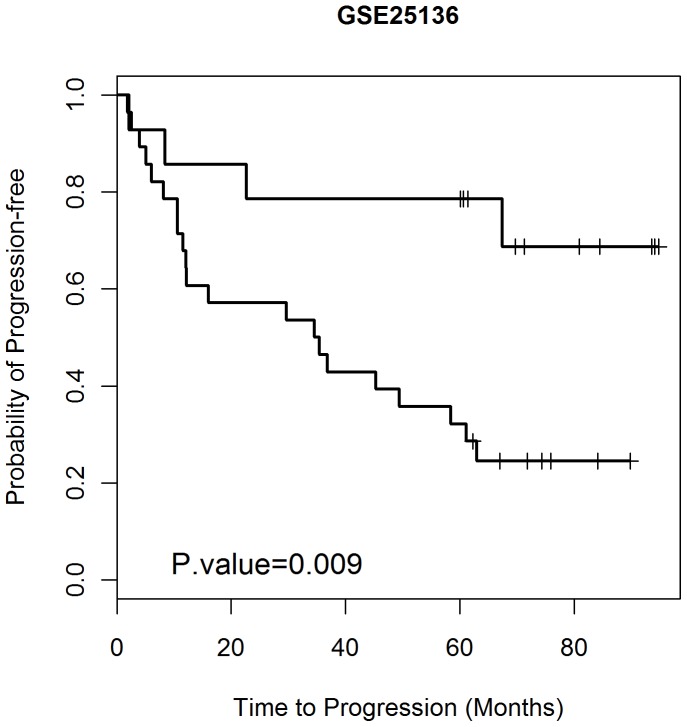
Survival analysis for the seven-gene Classifier with Gleason sum. Kaplan-Meier estimates of survival time of 42 independent patients in test Data Set 2 (GSE25136) according to the seven-gene Classifier with the Surgical Pathology-determined Gleason sum. The Gleason sum variable has the same weighting as each gene in the determination of classification.

We then examined whether any of the various clinical outcome values, Gleason score, PSA, age, volume, T stage, N stage, and M stage, had prognostic values that enhanced the performance of the classifier. The seven genes together with each clinical outcome were developed as new classifiers. In PAM analysis, the contributions of clinical outcome and seven genes are the evenly weighted. Only the post prostatectomy Gleason sum significantly improved the results with a substantial decrease of p value from 0.035 to 0.009 by the logrank test. The inclusion of Gleason sum with the seven-gene signature in the testing procedure using the independent Data Set 2 improved the accuracy and sensitivity to 74% and 84% for Data Set 2 (only Data Set 2 was used for this analysis due to the unavailability of Gleason sum for Data Set 3). Two more observed relapse patients were categorized into the high risk group. The Kaplan-Meier survival analysis (Figure 2) shows that the median survival of the patients in the high risk group defined by the seven-gene with post prostatectomy Gleason sum prognostic classifier was 34.6 months. 75% of patients in the high risk group had disease recurrence within 5 years, whereas 71% of patients in the low risk group remained relapse-free for at least 5 years. The estimated hazard ratio for the low risk and high risk group was 3.8 with a significant p-value of 0.009.

Finally we performed a multivariate Cox proportional hazards regression analysis of the prediction made by our classifier in combination with the clinical variables of age, pre-op PSA, pathological stage, and surgical margin but not with the Gleason sum which is included in our classifier. Only the p-value of the prediction by our classifier approached the significant level (p = 0.0686). The p-values of other ‘predictors’ are greater than 0.1. The results indicated that our classifier had better performance in risk stratification. We added this result to text on page 12–13. The result indicated that our classifier can better stratify risk.

### Validation of 23 Protein Expressing Genes of the 68 Tumor Gene Set

In order to validate the methods used here for the identification of tumor cell-specific expression, we compared the cell type specific expression found for RNA, i.e., *β_T_* and *β_S_*, with that observed for the respective protein expression in tumor and stroma cells provided by the Human Protein Atlas (HPA) as a test of whether the cell specific assignments of expression data were accurate. All 68 genes identified here as tumor cell specific were examined. We expected that the 68 genes identified here as tumor cell specific would exhibit protein expression that is more highly correlated with observed protein expression in tumor cells than in stroma cells. The protein expression profiling was carried out using the observed immunochistochemical (IHC) staining values observed in HPA as described (Materials and Methods). We collected data of 75 antibodies related to 49 of 68 tumor cell expressed genes (no antibodies for the remainder 19 genes) and then selected the 23 of the 49 genes that exhibited differentially expressed antibody intensities between normal subjects and prostate cancer patients for the correlation study. For each antibody, the protein expression level in tumor and stroma is averaged across the 12 patient samples. In all 672 IHC observations were used.

The RNA gene expression contribution from tumor and stroma was obtained from the CC analysis model for the 23 tumor genes. In the correlation study, we measured the two correlations: gene-protein expression correlation in tumor and gene-protein expression correlation in stroma. The results showed that the tumor correlation yielded a Pearson correlation coefficient of 0.41 with significant p value of 0.03 while the stroma correlation was insignificant with correlation of −0.02 (p value of 0.92). For comparison, a recent review paper [24] describing the correlation between protein and gene expression for various organisms including human showed that the correlation of 0.41 is comparable to the highest correlation observed for *homo sapiens* (0.46, p<0.001). Figure S2 shows a scatterplot of protein expression *versus* gene expression of our data. The correlation study demonstrates that the 23 informative genes identified by our proposed CC analysis model are indeed accurately identified as tumor cell expressed genes.

## Discussion

We hypothesized that more reliable cancer classifiers may be identified if cell-type heterogeneity was taken into account. We have developed a novel Cluster-Correlation analysis where the variation caused by cell-type distribution is controlled through multiple linear regression (MLR). The proposed CC analysis is a new gene differential expression analysis. There are two major features of the analysis (Figure S1). First, we incorporated known cell-type percentage into the analysis, avoiding false identification merely caused by varied cell type composition between tissue samples. Second, we performed unsupervised clustering, avoiding direct use of the biochemical recurrence information which is often not definitive due to data censoring. The two exclusive features make CC analysis better than traditional gene expression analyses. In a previous study [17] we compared the CC analysis model with traditional gene differential expression analyses such as by SAM and LIMMA. The simulation results showed that the new model outperformed the traditional gene differential expression analyses in terms of sensitivity and specificity. In addition, when these methods were applied to prostate cancer data, the CC analysis can identify genes that are significantly enriched or associated with prostate cancer related pathways such as the wnt signaling pathway, ECM-receptor interaction, focal adhesion and TGF- *β* signaling pathway [17].

By using the CC analysis model, we identified 68 tumor cell expressed genes treated as candidate clinical biomarkers for further investigation. The seven most significant tumor cell expressed genes were identified by analyzing tumor cell enriched samples using SAM. These seven genes were used in PAM to form a classifier, which was subsequently validated on two independent Data Sets. For these tests, we utilized test samples with >50% tumor cell content as estimated by CellPhred. It is impossible to get pure tumor samples due to the cell type heterogeneity intrinsic to most Gleason histology patterns and due to varying degrees of stroma and other elements with tissue samples selected for microarray analysis of “tumors”. By comparing the prediction accuracy of selected samples with various tumor cell percentages (samples with >10% tumor cell to >50% tumor cell), we determined that the best prediction was obtained when the tumor cell percentage of a given sample was greater than 50%. Therefore, the accuracy, sensitivity, and specificity of our independent testing result is likely an *underestimate* of the performance that would be obtained using for purer tumor samples.

The major limitation of most previous biomarker detection studies is that a single clinical Data Set was used for both signature discovery and validation. Recently, the first study to perform signature discovery and validation on independent data [25] used a recurrence algorithm that resulted in a sensitivity of 68%. The sensitivity was improved by incorporating PSA but only if the segregation of relapse and non-relapse subgroups was defined in the test data, which is similar to the strategy of previous studies – discovery and validation on the same clinical Data Set. In contrast, our seven-gene signature was first discovered by training data and validated on independent Data Sets.

To further assess the performance of our seven-gene signature, we carried out a PAM-based prediction comparison between our gene signature and other gene signatures identified in other studies. Table 2 shows the comparison of five different gene signatures – our seven-gene signature, the Bismar gene signature [26], and the Glinsky gene signatures 1–3 [25]. The results showed that our seven-gene signature provided the best accuracy and the best balance between sensitivity and specificity in independent tests.

In order to provide a comparison with an independent and accurate predictor, we also utilized a classification system CAPRA score [8] to determine the risk of recurrence for Data Set 1. The result showed that the accuracy of CAPRA score is only 54%, which is not comparable to the accuracy of our signature. This discrepancy may represent distinction in features of our population compared to the population used in the development of the CAPRA Score [8].

In conclusion, the seven-gene prognostic signature is closely associated with biochemical recurrence in patients after radical prostatectomy. This signature suggests practical applications such as stratification of patients according to risk in the trials of adjuvant treatment and identification of targets for the development of therapy for prostate cancer progression.

## Supporting Information

Figure S1 **Flow chart of the development of seven-gene classifier.**
(DOC)

Figure S2 **Protein expression versus RNA expression.** The RNA expression represents the RNA gene expression from tumor contribution.
(DOC)

Figure S3 **Boxplot of tissue composition for Data Set 1.**
(DOC)

Table S1 **Demographic characteristics of Data Set 1 and 2.**
(DOC)

Table S2 **Clinical and pathologic tumor characteristics of Data Set 1 and 2.**
(XLS)
