# Supplementary material for: An Accurate Prostate Cancer Prognosticator Using a Seven-Gene Signature Plus Gleason Score and Taking Cell Type Heterogeneity into Account
Source: PLoS One. 2012 Sep 28;7(9):e45178. doi: 10.1371/journal.pone.0045178 (PMC3460942; doi:10.1371/journal.pone.0045178)
Supplement: Figure S1 — Flow chart of the development of seven-gene classifier. (DOC) [file pone.0045178.s001.doc]

**Supporting Figure 1. Flow chart of the development of seven-gene classifier.**

22283 genes

324 genes

256 stroma genes

68 Tumor genes

7 Tumor genes

Cluster-Correlation analysis

SAM: Significance analysis of Microarray

Step 1: Unsupervised clustering with the inclusion of Multiple Linear Regression on cell type percentages

Step 2: Chi-square test on 2 x 2 contingency tables formed by cluster identity and observed relapse status.

Model selection: Two restricted models

Tumor gene: BIC_Tumor_ < BIC_Stroma_

Stroma gene: BIC_Tumor_ > BIC_Stroma_

BIC represents Bayesian information criterion.
