# Supplementary material for: An Accurate Prostate Cancer Prognosticator Using a Seven-Gene Signature Plus Gleason Score and Taking Cell Type Heterogeneity into Account
Source: PLoS One. 2012 Sep 28;7(9):e45178. doi: 10.1371/journal.pone.0045178 (PMC3460942; doi:10.1371/journal.pone.0045178)

**Supporting Figure 2. Protein expression versus RNA expression. The RNA expression represents the RNA gene expression from tumor contribution.**


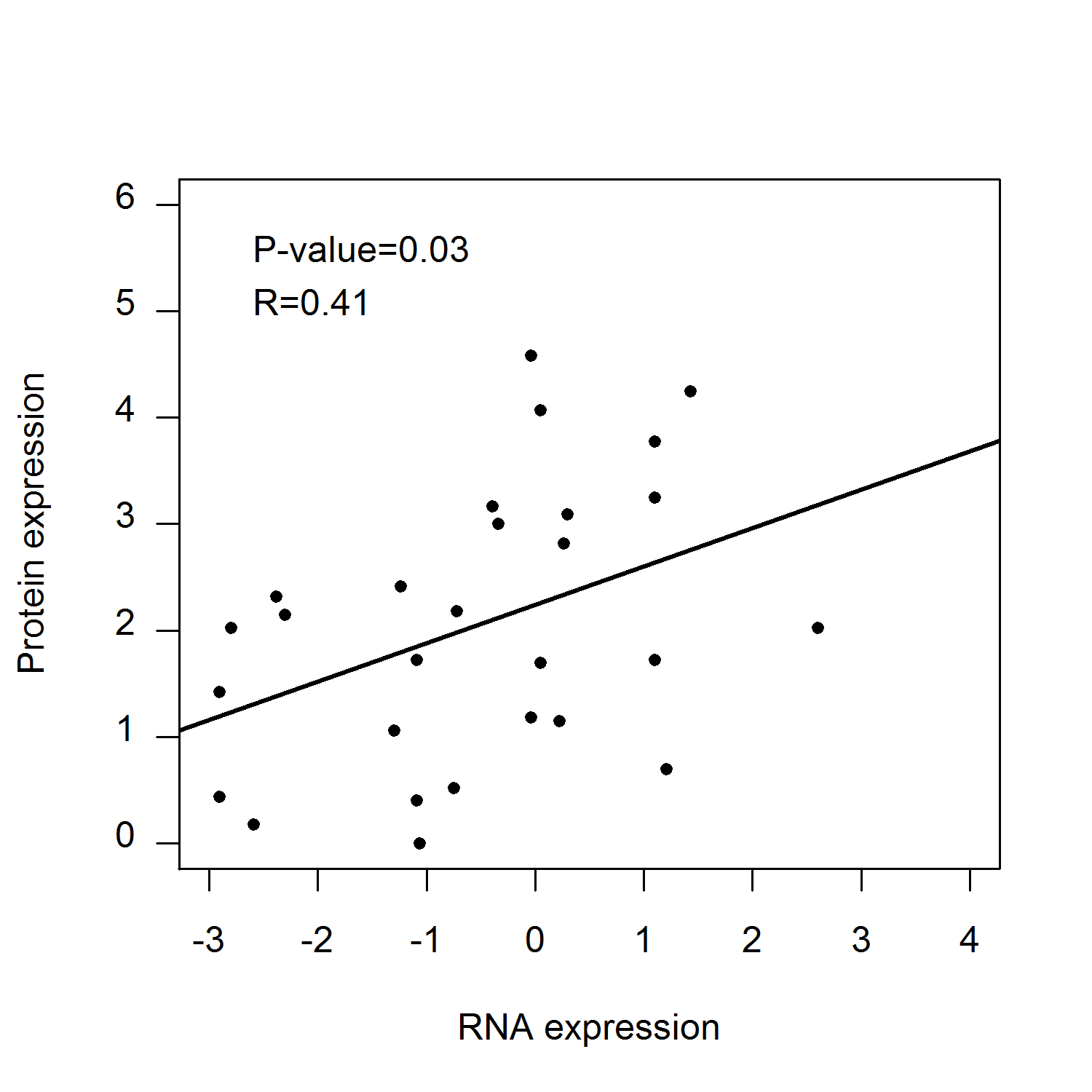

Supplement: Figure S2 — Protein expression versus RNA expression. The RNA expression represents the RNA gene expression from tumor contribution. (DOC) [file pone.0045178.s002.doc]
