# Supplementary material for: An Accurate Prostate Cancer Prognosticator Using a Seven-Gene Signature Plus Gleason Score and Taking Cell Type Heterogeneity into Account
Source: PLoS One. 2012 Sep 28;7(9):e45178. doi: 10.1371/journal.pone.0045178 (PMC3460942; doi:10.1371/journal.pone.0045178)
Supplement: Figure S3 — Boxplot of tissue composition for Data Set 1. (DOC) [file pone.0045178.s003.doc]

**Supporting Figure 3. Boxplot of tissue composition for Data Set 1.**
