# Supplementary material for: An Accurate Prostate Cancer Prognosticator Using a Seven-Gene Signature Plus Gleason Score and Taking Cell Type Heterogeneity into Account
Source: PLoS One. 2012 Sep 28;7(9):e45178. doi: 10.1371/journal.pone.0045178 (PMC3460942; doi:10.1371/journal.pone.0045178)
Supplement: Table S1 — Demographic characteristics of Data Set 1 and 2. (DOC) [file pone.0045178.s004.doc]

Table S1. Demographic characteristics of Data Set 1 and 2.

|  | **Data Set 1** | **Data Set 2** |
| --- | --- | --- |
| No. of patients | 78 | 79 |
| Age (years), mean (SD) | 62.5 (7.3) | 60.6 (6.2) |
| Preoperative PSA (ng/ml) | 9.5 | 11.2 |
| Average Follow up (Months) | 22.2 | 51.5 |
| Biochemical recurrence |  |  |
| Relapse | 45 (57.7%) | 37 (46.8%) |
| Non-relapse | 33 (42.3%) | 42 (53.2%) |
| Gleason sum |  |  |
| 3-6 | 30 (38.5%) | 17 (21.5%) |
| 7 (3+4) | 30 (38.5%) | -- |
| 7 (4+3) | 5 (6.4%) | -- |
| 7 | 35 (44.9%) | 44 (55.7%) |
| 8-10 | 13 (16.7%) | 18 (22.8%) |
| Pathological Stage |  |  |
| T1 | 2 (2.6%) | 34 (43.0%) |
| T2 | 47 (60.3%) | 43 (54.5%) |
| T3 | 26 (33.3%) | 2 (2.5%) |
| T4 | 3 (3.8%) | 0 (0.0%) |
| Surgical margins |  |  |
| Negative | 47 (60.3%) | 29 (36.7%) |
| Positive | 25 (32.1%) | 50 (63.3%) |
| Reference | GSE8218 | GSE25136 |
